# A triple-guidewire technique to stabilize the endoscope during endoscopic ultrasonography-guided pancreatic drainage following pancreaticoduodenectomy

**DOI:** 10.1055/a-2767-0864

**Published:** 2026-01-28

**Authors:** Shinichi Hashimoto, Makoto Hinokuchi, Satoshi Mori, Yu Yamazato, Yuji Tabira, Issei Kojima, Shiroh Tanoue

**Affiliations:** 1 12851 Digestive and Lifestyle Diseases, Kagoshima University Graduate School of Medical and Dental Sciences, Kagoshima, Japan

Endoscopic ultrasound-guided pancreatic duct drainage (EUS-PD) is challenging in patients with gastropancreatic anastomosis because the remnant pancreatic duct (PD) is typically short and tortuous for devices to be advanced through, and the endoscope is often unstable during the intervention. The double-guidewire technique for EUS-PD was therefore developed to stabilize the endoscope and prevent guidewire dislodgement during such procedures 1 2 . We present a case in which triple-guidewire placement in the PD proved useful for maintaining additional endoscopic stability during EUS-PD.

The patient was a 36-year-old man who had undergone pancreaticoduodenectomy with gastropancreatic anastomosis 5 months earlier for duodenal stenosis caused by annular pancreas. Three months postoperatively, the patient returned with left back pain and elevated serum amylase levels. Abdominal computed tomography (CT) showed dislodgement of the PD tube that had been placed, which had also dilated because of obstruction at the gastropancreatic anastomosis ( Fig. 1 ). EUS-PD was performed under intravenous anesthesia to treat the obstruction ( Video 1 ). The PD near the anastomosis was punctured using a 19 G needle. However, after the gastropancreatic fistula was dilated, a sudden pain response from the patient caused the guidewire to become dislodged. We re-punctured the pancreatic tail portion of the PD ( Fig. 2 **a** ) and advanced an initial 0.025-inch standard guidewire into the PD. To mitigate another movement-induced dislodgment, a second 0.025-inch hard-type guidewire was inserted into the duct using a double-lumen catheter. The third 0.035-inch guidewire was advanced into the stomach via the gastropancreatic anastomosis. The second guidewire was also advanced into the stomach ( Fig. 2 **b** ), improving the endoscope’s stability ( Fig. 2 **c** ). A 7 Fr plastic stent was then successfully placed in the PD, through the fistula ( Fig. 2 **d** ). The patient experienced no postoperative adverse events and was discharged 5 days post-procedure.

**Fig. 1:**
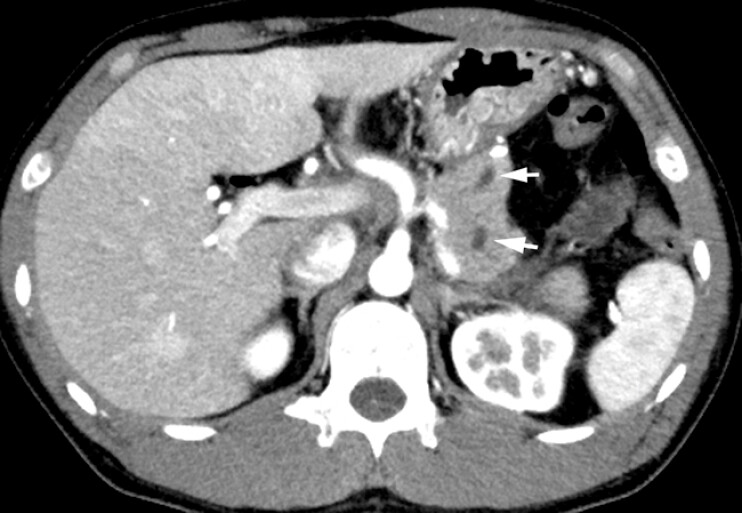
Computed tomography shows obstruction at the gastropancreatic anastomosis, as well as pancreatic duct dilatation (arrows).

**Fig. 2:**
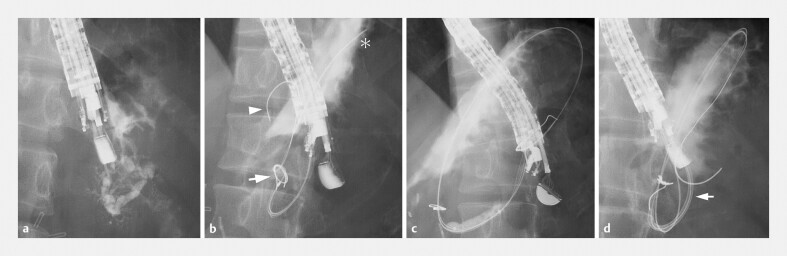
**a** Pancreatography following puncturing of the pancreatic tail reveals a short, tortuous pancreatic duct. **b** The initial 0.025-inch guidewire (arrow) is placed in the pancreatic duct. The second 0.025-inch guidewire (arrowhead) and the third 0.035-inch guidewire (asterisk) are then advanced into the stomach via the gastropancreatic anastomosis. **c** The three guidewires maintain the stability of the endoscope during dilation of the gastropancreatic fistula. **d** The plastic stent (arrow) on the second guidewire is advanced along with the other guidewires.

The use of a triple-guidewire approach during endoscopic ultrasound-guided pancreatic drainage successfully stabilized the endoscope.Video 1

Endoscopy_UCTN_Code_TTT_1AS_2AI

## References

[1] Hashimoto S Yamauchi T Hamada T A double-guidewire technique for endoscopic ultrasonography-guided pancreatic drainage after pancreaticoduodenectomy Endoscopy 2022 55 E270 E271 10.1055/a-1974-9436 36427497 PMC9831770

[2] Ogura T Ueno S Okuda A Technical feasibility and safety of a novel device for simultaneous double guidewire insertion and tract dilation during interventional EUS Dig Dis Sci 2025 70 4229 4234 10.1007/s10620-025-09250-1 40711744

